# Banker Plant Bonuses? The Benefits and Risks of Including Brassicas in Field Margins to Promote Conservation Biocontrol of Specialist Pests in Oilseed Rape

**DOI:** 10.3390/insects14040349

**Published:** 2023-03-31

**Authors:** Matthew. P. Skellern, Suzanne J. Clark, Andrew W. Ferguson, Nigel P. Watts, Samantha M. Cook

**Affiliations:** 1 Rothamsted Research, Harpenden, Hertfordshire AL5 2JQ, UK; 2 Limewood Science, The Paddock, Stainton by Langworth, Lincoln LN3 5BL, UK

**Keywords:** agri-environment schemes, regenerative agriculture, *Brassicogethes aeneus*, *Brassica napus*, rapeseed, *Meligethes*, *Ceutorhynchus*, *Dasineura*, ecosystem services

## Abstract

**Simple Summary:**

Insect pest management in oilseed rape (OSR) is challenging for farmers due to the lack of effective insecticides, so conservation biocontrol—reliance on natural pest control and management methods to support populations of the natural enemies of crop pests—is part of the solution for a greener future. Sown wildflower field margins are known to benefit many natural enemies of crop pests. However, the most effective natural enemies of OSR pests are parasitoids, which specialise in attacking these pests, which in turn specialise on Brassica host plants (including OSR), but few field margin mixtures contain brassicas. We screened six different species in the field to find the plants with the best potential as ‘banker plants’ to support parasitoid populations whilst not exacerbating the pests. Forage rape was the best ‘all-rounder’; supporting good numbers of pollen beetle parasitoids and also supporting parasitoids of weevil and brassica pod midge pests. Careful consideration of the brassica component of field margin mixtures could extend functionality towards enhanced biocontrol of specialist pests.

**Abstract:**

European agri-environment schemes include the use of flower-rich field margins to promote on-farm biodiversity, but species mixtures rarely include Brassicaceae. As pests of oilseed rape (OSR; *Brassica napus*) and their parasitoids are mostly brassica specialists, including brassica ‘banker plants’ in the mixtures would help support these important biocontrol agents and improve pest control throughout the crop rotation. We assessed the potential of six brassicaceous plants (replicated plots grown in the field) to enhance populations of parasitoids of OSR pests whilst minimising proliferation of their pest hosts. Fodder radish (*Raphanus sativus*) facilitated high production of parasitoids of the pollen beetle pest (*Brassicogethes aeneus*) but may proliferate *Ceutorhynchus* weevil pests due to low parasitism. Turnip rape (*B. rapa*) and the *B. rapa* hybrid ‘Tyfon’ showed potential to perform a trap cropping function for pests, but their early flowering phenology resulted in *B. aeneus* larvae escaping parasitisation, potentially assisting proliferation of this pest. Forage rape *B. napus* exhibited similarly high *B. aeneus* parasitoid production characteristics to *R. sativus* but did not potentiate problems with other pests, indicating that it would be a favourable banker plant option. Careful selection of plants in field margin mixtures is therefore needed to maximise their benefits and ideally the whole crop pest-beneficial complex needs to be studied, as focus on a single major pest risks unintended consequences with other pest problems.

## 1. Introduction

Agricultural intensification has led to fragmentation of semi-natural habitats within the farmed landscape [1], leading to a substantial loss of biodiversity from arable ecosystems [2,3,4]. As a result, concerns have been raised about the deterioration of important ecosystem services, including natural pest control [5].

Semi-natural habitats, which include extensive/calcareous grasslands, woodlands, hedgerows and grass or wildflower field margins, represent zones of relative stability compared with the frequently disturbed cropped environment [6] and often provide the natural enemies of crop pests with overwintering sites [7,8,9], floral resources [10,11], and alternative host or prey insects [12]. Consequently, these habitats often act as a source from which natural enemies colonise fields early in the growing season [13], but may also function as sinks, particularly later in the season when within-field resources become depleted, leading to a ‘spillover’ effect [14]. In recognition of these functions and in response to increasing pressures to develop alternative pest management strategies and also to support farmland biodiversity, remediation measures, often involving the establishment of field margins, have become an important part of European agri-environment schemes [15,16]. So far, field margin seed mixtures have tended to be designed for a specific purpose, for example to encourage pollinators [17,18], or to support farmland birds [19]. From a natural pest control perspective, grassy margins, which support cereal aphids and their natural enemies, have become an important area of study [20,21]. The need to design multi-functional margins increasing multiple services such as biological control and pollination has now been recognised [22]. Whilst the selection of plants providing floral resources to beneficial insects is an important part of this work, those promoting pest species should be avoided [23]. Sown ‘banker plants’ are potentially another important component of margin design, and although these systems have so far mostly been implemented on indoor crops (see [24] for a review), a few examples of research into outdoor systems exist [25,26]. Banker plants typically harbour non-pest herbivores, which serve as alternative hosts or prey for generalist parasitoids or predators of target pest species [24]. The use of plants harbouring non-pest herbivores eliminates possible exacerbation of pest populations, but where the important natural enemies of a pest are specialists, plants which support pest species must be considered; in this instance the balance must be towards natural enemies (and not pest) production. From a wider ecological perspective, systems promoting specialist rather than generalist natural enemies may indeed be desirable as detrimental spillover predation or parasitism effects [27] on non-target species in surrounding habitats are avoided. Ideally, these plants might also perform a trap-cropping function within the wider landscape, arresting the movement of pest populations onto crops during vulnerable growth stages [28,29,30,31]. Here, we assess the risks and benefits of such an approach, using four specialist pests of oilseed rape (*Brassica napus* L.) and their parasitoids, which have a high degree of host specificity, as a model study system.

Oilseed rape (OSR; *Brassica napus*) is an important source of plant-derived oil and protein, with an estimated global production of 72.4 million tonnes in 2020 [32]. Recent demand for biofuel use [33] has led to the crop occupying an increasingly significant position within arable rotations. In Europe, the pollen beetle, *Brassicogethes aeneus* F. (syn *Meligethes aeneus*) (Coleoptera: Nitidulidae), an inflorescence pest, causes significant damage to the crop and is considered to be the main pest of the flowering stage. Two pests of the developing pods, the cabbage seed weevil *Ceutorhynchus obstrictus* Marsh. (syn. *C. assimilis* Payk) (Coleoptera: Curculionidae) and the pod midge *Dasineura brassicae* Winn. (Diptera: Cecidomyidae) also cause yield losses, along with the stem-tunnelling larvae of the cabbage stem weevil *Ceutorhynchus pallidactylus* Marsh. [34].

The parasitoid complexes considered important for the biocontrol of these pests are given in Table 1. Parasitoids of pollen beetles, pod midge and cabbage stem weevil (in this latter case, a single species) all overwinter as diapausing adults in the soil of former OSR fields and then emerge in the following spring, predisposing them to substantial tillage-related mortality [35,36]. Those attacking cabbage seed weevil, however, are unaffected by tillage as they pupate within the pods and emerge before harvest, overwintering in sheltered places including evergreen foliage [37]. All attack the egg/larval stages.

The inclusion of brassicas within field margins which are less disturbed (in terms of cultivation) than the crop area may particularly benefit the tillage-susceptible brassica specialist parasitoids and support their populations throughout the arable rotation. This may be an especially important function in landscapes where the spatial separation of large blocks of contiguous OSR fields forces the parasitoids, which have weaker dispersal abilities than their hosts [38], to migrate large distances from their emergence sites. Using field experiments, we assessed six different species of the Brassicaceae as potential banker plants, in terms of their ability to enhance populations of brassica specialist parasitoids, versus the risk that they will exacerbate pest populations.

**Table 1:** The main Hymenopteran parasitoid species specialising on insect pests of oilseed rape in the study system.

Pest	Parasitoids	Notes and References
Pollen beetle (*Brassicogethes aeneus*)	*Tersilochus heterocerus* (Thomson) ^1,a,†^ *Phradis interstitialis* (Thomsson) ^1,a,†^ *Phradis morionellus* (Holmgren) ^1,a,†^	*Diospilus capito* (Nees) ^2,a,†^ and *Blacus nigricornis* Haeselbarth ^2,a,†^ are widespread but less common and therefore of minor importance for biocontrol [37]
Cabbage seed weevil (*Ceutorhynchus obstrictus*)	*Trichomalus perfectus* Walker ^3,b,†^ *Stenomalina gracilis* Walker ^3,b,†^ *Mesopolobus morys* Walker ^3,b,†^	[37,39]
Brassica pod midge (*Dasineura brassicae*)	*Platygaster subuliformis* Kieffer ^4,a,‡^ *Omphale clypealis* Haliday ^5,a,†^	[37,40]
Cabbage stem weevil (*Ceutorhynchus pallidactylus*)	*Tersilochus obscurator* Aubert ^1^	[41]

^1^ Ichneumonidae; ^2^ Braconidae; ^3^ Pteromalidae; ^4^ Platygastridae; ^5^ Eulophidae; ^a^ Endoparasitic (lay eggs inside host); ^b^ Ectoparasitic (lay eggs on the surface of host); ^†^ larval parasitoid; ^‡^ egg-larval parasitoid.

## 2. Materials and Methods

### 2.1. Plant Selection and Experimental Design

Six winter-sown brassicas were selected for testing: oilseed rape (OSR; *Brassica napus* L. subsp. Oleifera) cv Castille, fodder radish (*Raphanus sativus* L.) cv. Apoll, forage rape (*Brassica napus* L. subsp. Biennis) cultivars ‘Hobson’ and ‘Emerald’, stubble turnip rape (*Brassica rapa* L. Rapifera group) cv Jupiter, and Tyfon (a *B. rapa* L. Rapifera group x *B. rapa* L. Pekinensis group hybrid). Selection was based on commercial availability of winter annual cultivars commonly used in animal fodder mixtures, which were known to flower during spring (unpublished preliminary data) and a commonly used OSR crop cultivar as a ‘control’. Experimental plots (6 m × 6 m) spaced 12 m apart, were sown on 9 September 2010, on two fields at Rothamsted Research, Harpenden, UK, in a randomised complete block design with two blocks of the six treatments on each field (i.e., 4 treatment replicates in total). The experiment was sited in a crop of winter oilseed rape and the 12 m areas around each plot were mowed regularly to prevent flowering. Crop management was consistent for all plots and no insecticides were applied during the growing season. After harvest, the plots were kept fallow (i.e., no tillage or following crop) to enable collection of emerging parasitoids in the following spring.

### 2.2. Plant Growth Stage Assessments

Growth stages (GS) of plants in each plot were assessed at intervals of ≤7 days throughout March to July, following the BBCH scale of Lancashire et al. [42]. On each occasion, plots attaining the following thresholds were noted: ≥25% of plants at GS 51 (green bud), ≥25% of plants at GS 60 (first flowers open), ≥95% of plants at GS 69 (flowering completed). Thereafter, pod ripening was checked less regularly, primarily to determine GS 85 (seeds ripe, black and hard) i.e., the optimum stage for monitoring pests and parasitism at the pod stage (see Section 2.4), after which recording ceased.

### 2.3. Suction Sampling of Adult Insects

Vortis suction sampling [43] (Burkhard Manufacturing Co., Rickmansworth, UK) was conducted on each treatment plot on five occasions, monthly, from mid-March to mid-July 2011. Samples were taken from the plant canopy in a sweeping motion while walking along the perimeter of each plot (at a speed of approximately 1 ms^−1^). In this way, the plants were sampled in a c. 50 cm-wide strip along each side of the plot c. 50 cm into the plot from the plot edges. The samples were frozen for storage until the insects could be identified and counted in the laboratory. Parasitoids were identified using the key by Ferguson et al. [44].

### 2.4. Pod Infestation by the Pest Larvae of Cabbage Seed Weevil and Brassica Pod Midge and Parasitism of Cabbage Seed Weevil Larvae

Four pods (two from the primary raceme and two from the third highest order lateral raceme) were collected from 50 randomly chosen plants per plot when they reached GS 85. Collections were made from turnip rapes ‘Tyfon’ and ‘Jupiter’, and from fodder radish on 10 June, 14 June, and 14 July, respectively. Because of growth stage differences amongst plots, collections were split between 27 June and 8 July for plots of OSR and forage rape cv Emerald, and between 27 June and 1 July for forage rape cv Hobson plots. Pods were dissected and living, dead or parasitised cabbage seed weevil larvae, and larval/parasitoid exit holes were recorded along with those containing brassica pod midge larvae. Parasitoids of cabbage seed weevil are ectoparasitoids, so eggs/larvae are visible on the surface of the pest larvae; parasitoids of brassica pod midge are endoparasitoids and as determination of parasitism requires either larval dissection or rearing, was not carried out in this study.

### 2.5. Trapping of Emerging Insects

To assess the ‘production’ of parasitoids by the different Brassicaceae, an emergence trap was placed in the central position of each of the former brassica plots (i.e., following harvest of the plots, the ground was left uncultivated to allow any parasitoids which had dropped from the plants in the plots to complete their pupation and emerge from the fallow ground). Each trap enclosed an area of 0.5 m^2^ ground, and insects emerging from pupation were collected in a plastic jar containing 70% ethanol [45]. Traps were installed in February 2012, and samples were collected at three-weekly intervals during March–July, pooled, and remained stored in 70% ethanol until the insects were identified [44].

### 2.6. Statistical Analyses

All analyses were carried out using GenStat, version 15 (VSN International, Hemel Hempstead, UK). Season totals of insects from each treatment over the five suction sample dates, and pooled emergence trap collections, were each analysed using analysis of variance (ANOVA) with terms to account for field/block. The totals were transformed (log_10_) and if any zeros were present an offset of one was added to all totals before transformation. The similarly transformed monthly suction sample data were analysed using repeated measures ANOVA, in which each of the 24 plots of the original design were split for the five sample dates. Proportions of infested pods were analysed using logistic regression (generalised linear model with Binomial error and logit link), fitting brassica treatment effects after allowing for differences amongst blocks.

## 3. Results

### 3.1. Growth Stage Assessments

**Figure 1:**
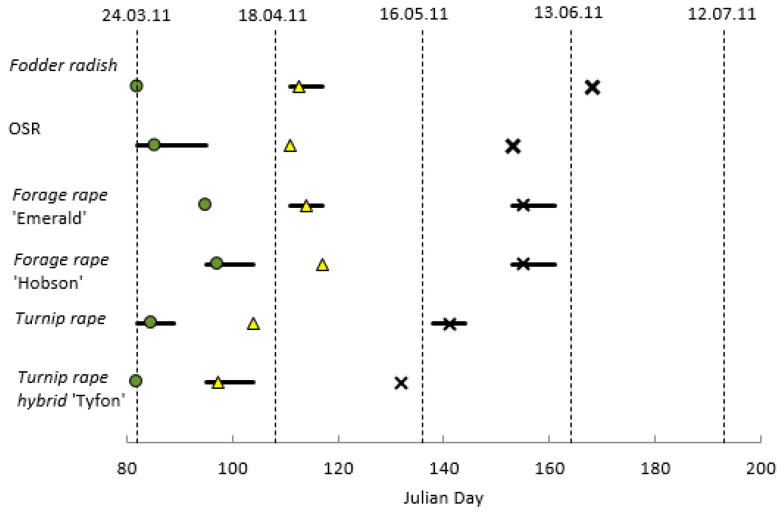
Key brassica plant growth stages relative to suction sample dates (dotted lines), averaged over four plots of each Brassicaceae treatment ^†^; horizontal bars represent the range of dates over which the key growth stages were attained. Filled green circles: ≥25% of plants at GS 51 (green bud); filled yellow triangles: ≥25% of plants at GS 60 (onset of flowering); crosses (x): flowering finished for ≥95% of plants. ^†^ Brassicaceae treatments: Fodder radish (*Raphanus sativus*) cv Apoll; Oilseed rape (OSR; *Brassica napus*) cv Castille; Forage rape (*Brassica napus*) cv Emerald and cv Hobson; Turnip rape (*Brassica rapa*) cv Jupiter and Tyfon (hybrid of *B. rapa* Rapifer group × *B. rapa* Pekinensis group).

All fodder radish and Tyfon plots reached GS 51 (green bud; flower buds just visible) by 24 March (the first suction sample), followed by plots of turnip rape, OSR and forage rape cv Emerald and cv Hobson, on average 2, 3, 13 and 15 days later, respectively (Figure 1). Only the turnip rape types were in flower on 18 April, and by 16 May all treatments were flowering, except for ‘Tyfon’, which had finished. Fodder radish had the longest duration of inflorescence emergence and flowering and was the only brassica to remain in flower on and slightly beyond 13 June.

### 3.2. Suction Sampling of Adult Insects

Season totals of all pests and parasitoids surveyed differed significantly amongst the brassica types, except for pollen beetle adults which were by far the most abundant insect found on all treatments (Table 2). Fodder radish hosted the highest abundance of pollen beetle parasitoids and the largest populations of cabbage stem weevil and its parasitoid (only *Tersilochus obscurator* was found). However, it accommodated the smallest numbers of cabbage seed weevil parasitoids, and brassica pod midge and its parasitoids. The turnip rape types hosted the lowest season totals of pollen beetle larval stages and parasitoids (Table 2). Furthermore, numbers of cabbage seed weevil and brassica pod midge adults were highest on ‘Tyfon’. The largest season totals of brassica pod midge parasitoids were found on the two forage rape cultivars (Emerald and Hobson) (Table 2). Regarding the species composition of parasitoids of each pest found on the different treatments, the parasitoid *Tersilochus heterocerus* Thoms. was the most abundant species which attacks pollen beetles to be found on the turnip rape types, while on the remaining brassica types *Phradis interstitialis* Thoms. was the most abundant species (Figure S1a). Parasitoids of cabbage seed weevil mainly comprised *Trichomalus* sp (Figure S1b), and *Platygaster subliformis* was most abundant parasitoid of brassica pod midge found in the suction samples although around a third of the sample comprised *Omphale clypealis* (Figure S1c).

**Figure 2:**
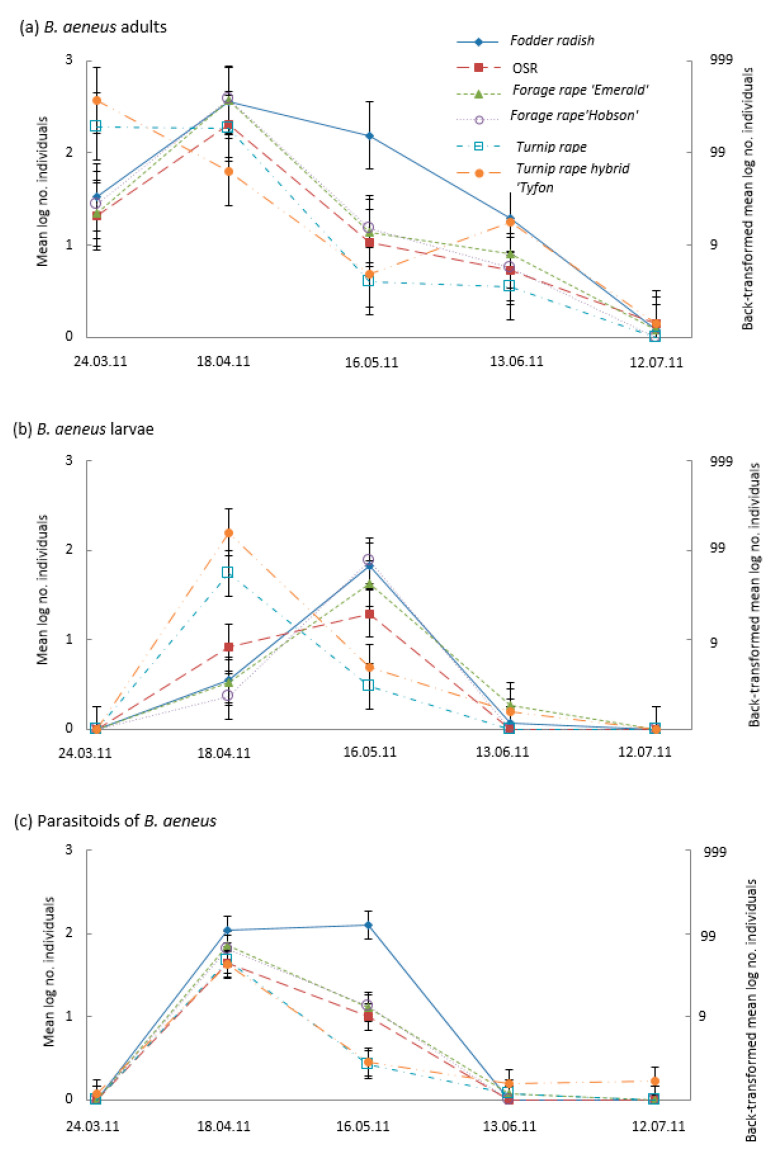
Mean numbers (log scale—left axis, and back-transformed -right axis) of pollen beetle (*Brassicogethes aeneus*) (**a**) adults, (**b**) larvae and (**c**) parasitoids in suction samples taken on five different dates from plots (*n* = 4) of different Brassicaceae: Fodder radish (*Raphanus sativus*) cv Apoll; Oilseed rape (OSR, *Brassica napus*) cv Castille; Forage rape (*Brassica napus*) cv Emerald and cv Hobson; Turnip rape (*Brassica rapa*) cv Jupiter and Tyfon (a hybrid of *B. rapa* Rapifer group × *B. rapa* Pekinensis group). Confidence intervals (CIs; vertical bars) are valid for within-date comparisons; for between-date comparisons, CIs should be based on SEM values of (**a**) 0.1711, (**b**) 0.1219 and (**c**) 0.0827.

Analyses of the monthly suction sample data indicated significant brassica type x time interactions (Table S1). Adult pollen beetle populations were present on the turnip rape types on the first assessment and declined early compared to other treatments, with the largest numbers of adults and larvae observed in the March and April samples, respectively (Figure 2a,b). Numbers of pollen beetle parasitoids on the turnip rape types peaked in April but declined more rapidly than on the other brassicas, resulting in these plants hosting the lowest parasitoid numbers in May (Figure 2c). The *B. napus* cultivars (OSR and ‘Hobson’ and ‘Emerald’ forage rapes) shared similar phenology of pollen beetle adults, larvae, and their parasitoids; adult and parasitoid numbers peaked in April and declined thereafter, whilst larval numbers peaked later in May (Figure 2a–c). Both pollen beetles and their parasitoids were retained in high numbers for longer on fodder radish than other treatments, being particularly apparent in the May sample (Figure 2a–c).

**Figure 3:**
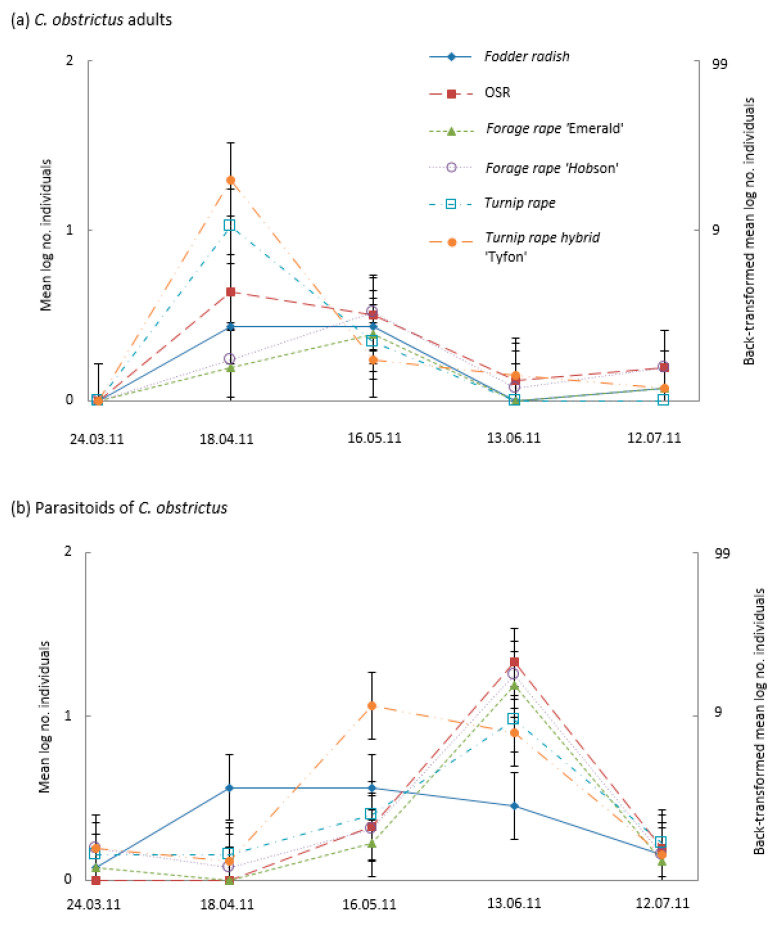
Mean numbers (log scale—left axis and back-transformed –right axis) of cabbage seed weevil *Ceutorhynchus obstrictus* (**a**) adults and (**b**) parasitoids in suction samples taken monthly from plots (*n* = 4) of different Brassicaceae: Fodder radish (*Raphanus sativus*) cv Apoll; Oilseed rape (OSR, *Brassica napus*) cv Castille; Forage rape (*Brassica napus*) cv Emerald and cv Hobson; Turnip rape (*Brassica rapa*) cv Jupiter and Tyfon (a hybrid of *B. rapa* Rapifer group × *B. rapa* Pekinensis group). CIs (vertical bars) are valid for within-date comparisons; for between-date comparisons, CIs should be based on SEM values of (**a**) 0.1022 and (**b**) 0.0999.

Cabbage seed weevil numbers exhibited large April peaks in the turnip rape cultivars (Figure 3a). Weevil numbers in OSR and fodder radish were comparatively lower, with peaks in April–May (Figure 3a). Weevil numbers on the forage rape cultivars peaked latest, in the May sample (Figure 3a). Numbers of cabbage seed weevil parasitoids peaked on most treatments in the June sample apart from fodder radish which hosted comparatively few parasitoids on this date (Figure 3b). Parasitoid numbers on turnip rape ‘Tyfon’ peaked earlier and were highest amongst the brassicas in the May sample (Figure 3b).

**Figure 4:**
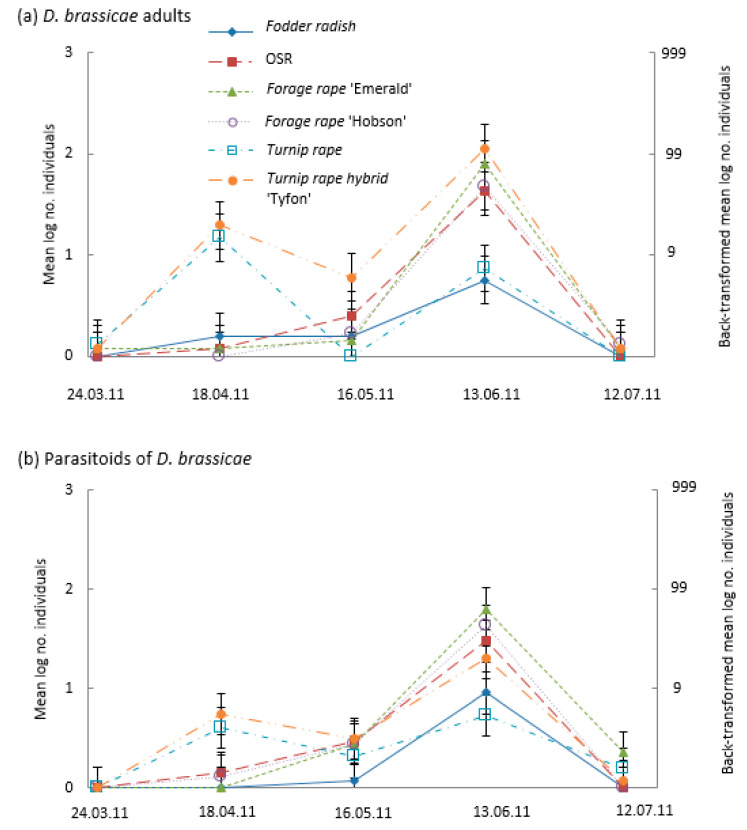
Mean (log scale—left axis and back-transformed -right axis) numbers of Brassica pod midge (*Dasineura brassicae*) (**a**) adults and (**b**) parasitoids in suction samples taken monthly from plots (*n* = 4) of different Brassicaceae: Fodder radish (*Raphanus sativus*) cv Apoll; Oilseed rape (OSR, *Brassica napus*) cv Castille; Forage rape (*Brassica napus*) cv Emerald and cv Hobson; Turnip rape (*Brassica rapa*) cv Jupiter and Tyfon (a hybrid of *B. rapa* Rapifer group × *B. rapa* Pekinensis group). CIs (vertical bars) are valid for within-date comparisons; for between-date comparisons, CIs should be based on SEM values of (**a**) 0.1169 and (**b**) 0.0974.

The phenology of both pod midge adults and their parasitoids on the turnip rape cultivars showed dual peaks (Figure 4a,b), the first in April, when these brassicas hosted the highest numbers, and the second in June, when numbers simultaneously peaked on the remaining brassicas.

**Figure 5:**
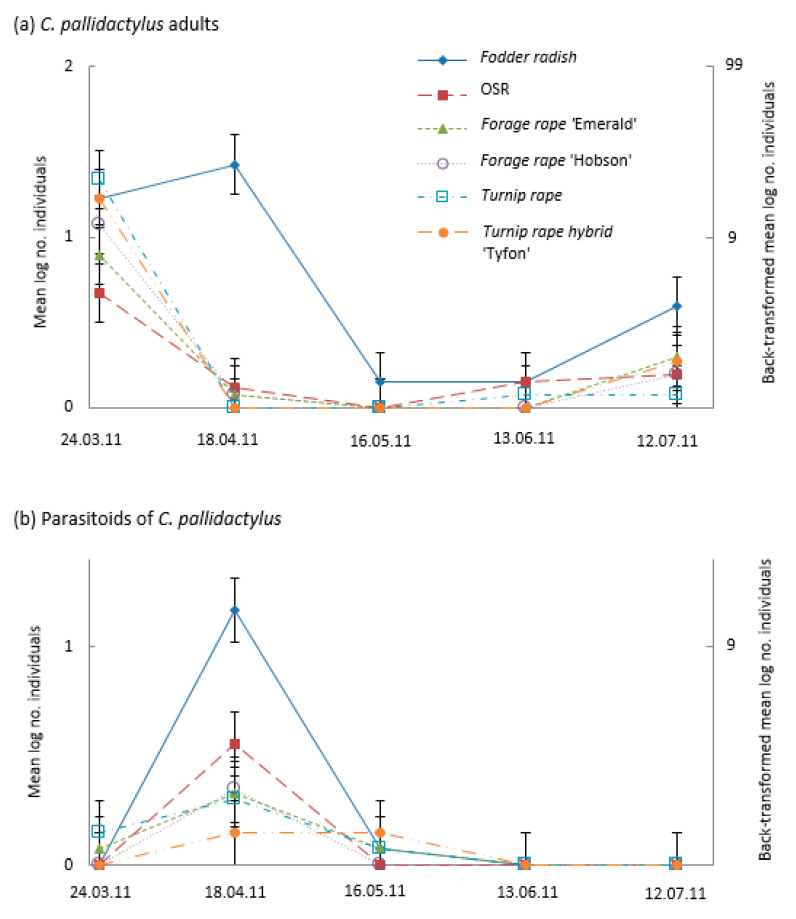
Mean numbers (log scale—left axis and back-transformed right axis) of cabbage stem weevil (*Ceutorhynchus pallidactylus*) (**a**) adults and (**b**) parasitoids in suction samples taken monthly from plots (*n* = 4) of different Brassicaceae: Fodder radish (*Raphanus sativus*) cv Apoll; Oilseed rape (OSR; *Brassica napus*) cv Castille; Forage rape (*Brassica napus*) cv Emerald and cv Hobson; Turnip rape (*Brassica rapa*) cv Jupiter and Tyfon (a hybrid of *B. rapa* Rapifer group × *B. rapa* Pekinensis group). CIs (vertical bars) are valid for within-date comparisons; for between-date comparisons, CIs should be based on SEM values of (**a**) 0.0806 and (**b**) 0.0723.

Cabbage stem weevil phenology was similar on all treatments (with the exception of fodder radish); the highest numbers were found in the March sample then declined into April and May, with numbers slightly increasing during June and July (Figure 5a). Cabbage stem weevil numbers were highest on fodder radish, with a large April peak and the largest late-season abundance in July (Figure 5a). Peak numbers of the cabbage stem weevil’s parasitoid (*T. obscurator*) occurred on all brassicas in April and were most abundant on fodder radish than on the other brassicas (Figure 5b).

### 3.3. Pod Infestation by the Pest Larvae of the Cabbage Seed Weevil (Ceutorhynchus obstrictus), the Brassica Pod Midge (Dasineura brassicae), and Parasitism of Cabbage Seed Weevil Larvae

**Figure 6:**
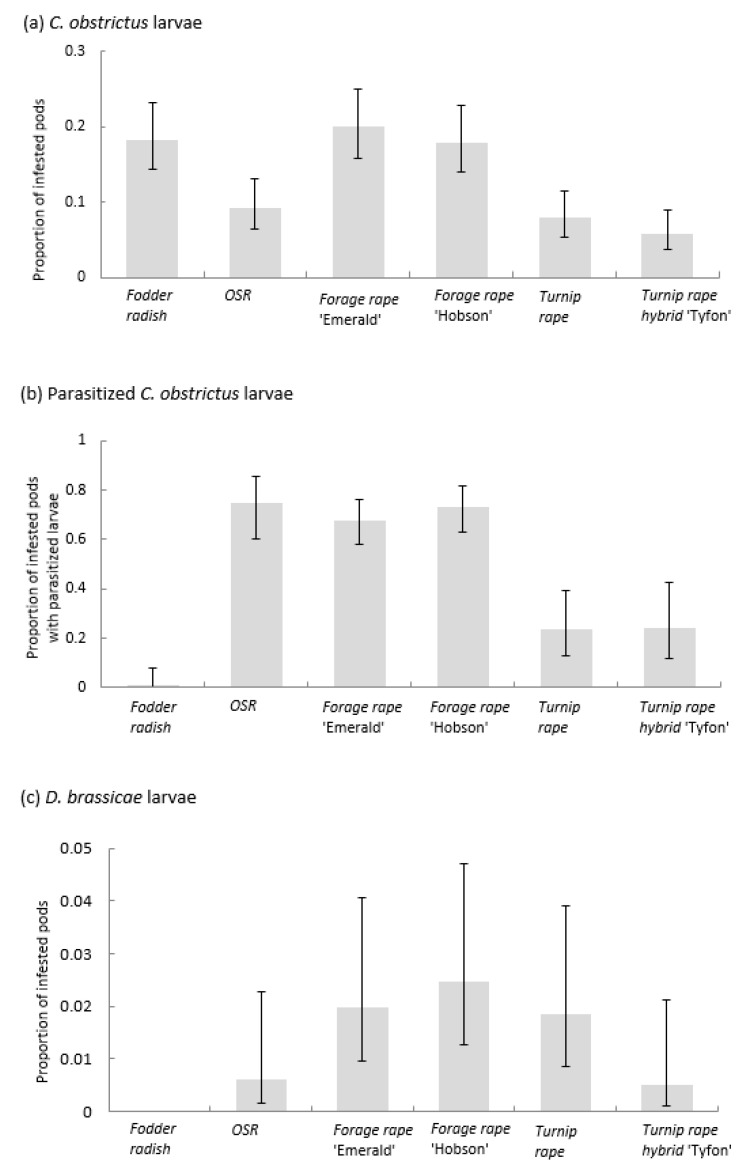
Proportion (back-transformed logistic regression predictions, ±CIs) of (**a**) pods infested by cabbage seed weevil (*Ceutorhynchus obstrictus*) larvae; (**b**) seed weevil-infested pods with parasitised larvae; (**c**) pods infested by brassica pod midge (*Daseneura brassicae*) larvae. Pods sampled from plots of different Brassicaceae: Fodder radish (*Raphanus sativus*) cv Apoll; Oilseed rape (OSR; *Brassica napus*) cv Castille; Forage rape (*Brassica napus*) cv Emerald and cv Hobson; Turnip rape (*Brassica rapa*) cv Jupiter and Tyfon (a hybrid of *B. rapa* Rapifer group × *B. rapa* Pekinensis group).

The proportions of pods infested by cabbage seed weevil larvae differed amongst brassica types (F_5,15_ = 12.58, *p* < 0.001; average LSD = 0.517; Figure 6a). Fodder radish and both forage rape cultivars (‘Emerald’ and ‘Hobson’) had similar proportions of infested pods (18.3%, 20.0% and 17.9%, respectively); the proportions for OSR, turnip rape cv Jupiter the turnip rape hybrid Tyfon were similar to one another but lower overall (9.2%, 7.9% and 5.7%, respectively).

Proportions of cabbage seed weevil-infested pods containing parasitised larvae also differed amongst the brassicas (F_5,15_ = 39.77, *p* < 0.001; average LSD = 1.269; Figure 6b). Parasitism of larvae in OSR and both forage rape cultivars (‘Emerald’ and ‘Hobson’) was high and similar (74.7%, 67.9% and 73.5%, respectively) whilst parasitism of larvae in pods of turnip rapes ‘Tyfon’ and ‘Jupiter’ was lower at 24.0% and 23.7%, respectively. The proportion of parasitised larvae from fodder radish pods was very low, at only 1.3%.

The proportions of pods infested by pod midge larvae differed amongst the brassicas (F_5,15_ = 4.31, *p* = 0.012; Figure 6c). Infestation rates were similar in both forage rapes (‘Hobson’ and ‘Emerald’) and in *B. rapa* ‘Jupiter’ (2.5%, 2.0 and 1.8%, respectively) but infestation was lower on OSR (0.6%) and ‘Tyfon’ (0.5%). No infested fodder radish pods were found.

### 3.4. Parasitoid Emergence from Emergence Traps

**Figure 7:**
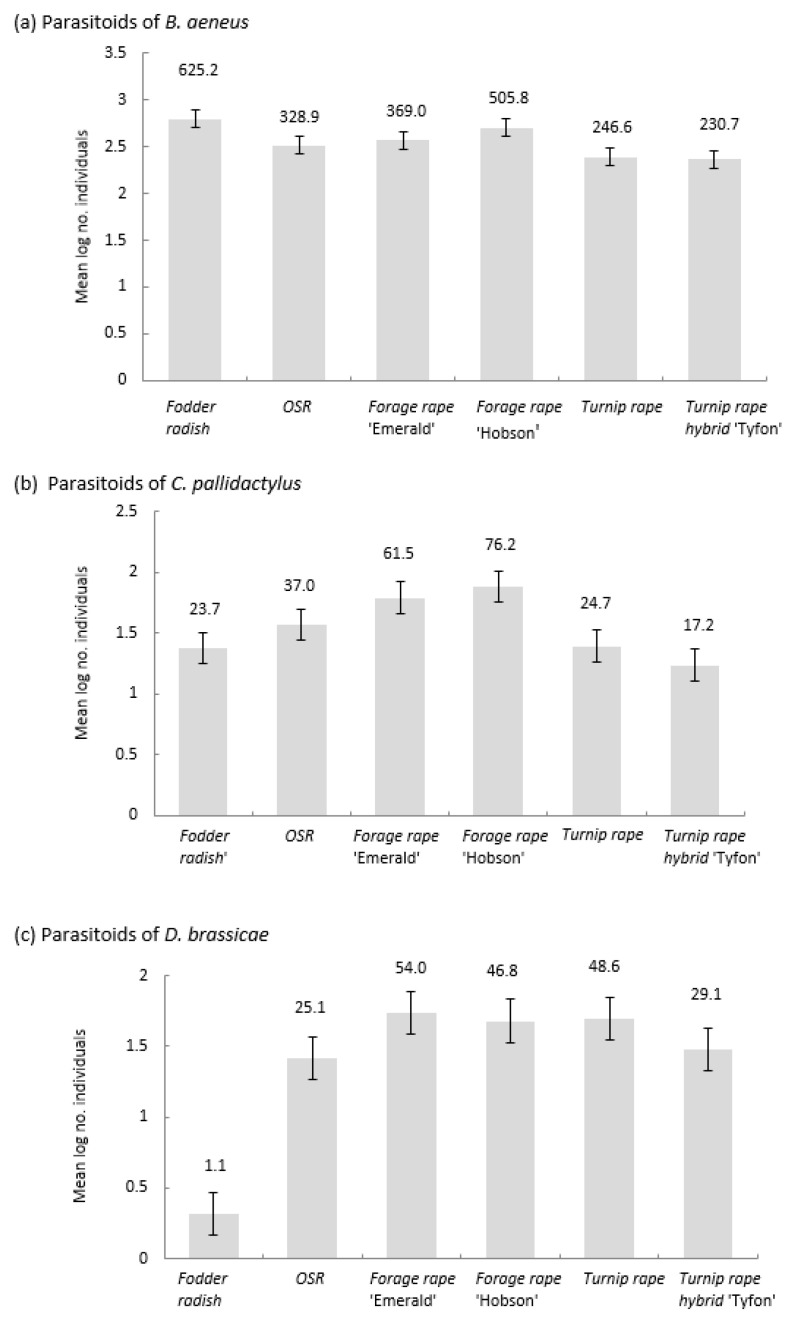
Mean number (log scale, ± SEM) of parasitoids of (**a**) Pollen beetle (*Brassicogethes aeneus*), (**b**) Cabbage stem weevil (*Ceutorhynchus pallidactylus*), and (**c**) Brassica pod midge (*Dasineura brassicae*) captured emerging from the site of different Brassicaceae plots (*n* = 4): Fodder radish (*Raphanus sativus*) cv Apoll; Oilseed rape (OSR; *Brassica napus*) cv Castille; Forage rape (*Brassica napus*) cv Emerald and cv Hobson; Turnip rape (*Brassica rapa*) cv Jupiter and Tyfon (a hybrid of *B. rapa* Rapifer group × *B. rapa* Pekinensis group). Back-transformed values are shown above each bar.

*Phradis interstitialis* was the most abundant parasitoid of pollen beetles emerging from former plots of all brassica types, but *T. heterocerus* made up large proportions of the individuals emerging from the turnip rape types (Figure S2a). Emerging parasitoid totals differed amongst the brassicas (F_5,15_ = 2.54, *p* = 0.002, LSD = 0.2023; Figure 7a). More individuals emerged from fodder radish than from the other brassicas, except for forage rape cv Hobson. Emerging parasitoid numbers were lowest for the turnip rape types.

*Tersilochus obscurator* was the only species parasitising cabbage stem weevil found in the emergence trap samples, and its abundance differed significantly between the brassica types (F_5,15_ = 7.47, *p* = 0.001, LSD = 0.2796; Figure 7b); most individuals emerged from the forage rape plots, and fewest from ‘Tyfon’, followed by fodder radish and turnip rape ‘Jupiter’.

Numbers of emerging parasitoids of the brassica pod midge (mainly *P. subliformis*; Figure S2b) differed amongst the brassicas (F_5,15_ = 25.24, *p* < 0.001, LSD = 0.3250; Figure 7c). Fewer parasitoids emerged from fodder radish plots than from the other brassicas.

## 4. Discussion

Flower mixtures used in sown field margins in agro-ecosystems are often designed to generally enhance biodiversity, making them inefficient for ecosystem service provision in adjacent crops. Although some positive effects of ‘general’ flower mixtures have been found on biocontrol in the crop [46,47,48], effects are generally restricted to generalist natural enemies and are not optimised to attract the main natural enemies in a system [49]. Banker plants that support the most important natural enemies of crop pests could be added to flower seed mixtures included in agri-environment schemes, creating a more efficient mixture for natural pest control, especially when specialist natural enemies are of importance. Oilseed rape (OSR) is affected by several pests which are specialist on Brassicaceae [34] and these pests, in turn are regulated by specialist parasitoids [37]. We screened six different plant species of the Brassicaceae to assess the best option as a banker plant to support parasitoids OSR pests without exacerbating the pests. We assessed abundance of adult pests and their parasitoids on the different plants, parasitisation and compared production of new-generation parasitoids emerging from plots sown to the different treatments. Different pests studied responded differently to the different plant species tested (summarised in Table S2). We found that selecting an optimal combination of field margins plants can be difficult to achieve without incurring negative consequences. For example, our results pointed to fodder radish (*Raphanus sativus*) as the most useful plant for attracting and producing parasitoids of pollen beetle (*Brassicogethes aeneus*) but, conversely, it contributes little to maintaining brassica pod midge (*Dasineura brassicae)* parasitoids and could potentially assist the proliferation of brassica specialist weevil pests. Whilst other studies have highlighted the need for caution in field margin plant selection because of floral resource-mediated positive influences on target pest species [23,50], the present study illustrates an alternative host plant-mediated situation where there are simultaneous positive and negative effects on different pest species of the same crop. Our study serves as a reminder of the perils of studies that focus on only one (often the primary) pest of a given system; potential plant composition effects on all pests relevant to a rotation should therefore be considered wherever possible.

### 4.1. Banker Plants for Parasitoids of Pollen Beetles

Fodder radish attracted the most pollen beetles (*B. aeneus*) and their parasitoids, and also produced the highest numbers of their parasitoids; numbers were lowest on OSR and Tyfon (summarised in Table S2). The presence of pollen beetle adults and the beetle’s parasitoids was extended on fodder radish relative to the other brassicas, reflecting the plant’s prolonged flowering period (and the known attraction of beetles to flowering stages of OSR [51,52]). The presence of pollen beetle larvae on fodder radish, however, was not similarly extended, suggesting that the fecundity of the pollen beetle populations maintained later into the season on fodder radish was low. Pollen beetles can reduce egg production [53] and egg size [54] on less acceptable host plants, and these factors may help explain the incongruence of adult and larval phenology on fodder radish. Alternately, larval development may have been poor on this host plant. Indeed, due to observations of poor larval development, this species has been proposed as a potential ‘dead end trap crop’ for pollen beetles [30]. Poor larval performance, however, would not necessarily preclude the high rates of parasitoid return observed in our study if parasitism rates were particularly high. Indeed, Billqvist and Ekbom [55] observed high larval parasitism rates on another low quality host plant, white mustard (*Sinapis alba*), possibly because increased mobility of ‘uncomfortable’ larvae on these plants leads them to greater parasitoid exposure, and this brassica also has been suggested as a useful field margin plant to help promote pollen beetle parasitoid populations [38].

### 4.2. Banker Plants for Parasitoids of Weevil Pests

Adult parasitoids of cabbage seed weevils (*Ceutorhynchus obstrictus*) captured in suction samples and new-generation individuals emerging from pupation were highest on the *Brassica* species tested (OSR and forage rapes, respectively). The vast majority of cabbage seed weevil larvae on fodder radish escaped parasitism as reflected in the very low rate of 1.3%, compared with parasitism rates of c. 20% for the turnip rape types and c. 70% for the *B. napus* types. This is in contrast to Kovács et al. [31], who found 90–94% parasitism of cabbage seed weevil larvae on fodder radish, but this rate was not significantly greater than on other species studied (including OSR), and populations of cabbage seed weevils were extremely low in their study; furthermore, their study was performed on spring-sown cultivars (not winter sown as in the present study). It is possible that the thickened, spongy pod wall of fodder radish provides a protective environment for cabbage seed weevil larvae, but this requires further research. Low parasitism rates on fodder radish, coupled with the fact that pod infestation rates by cabbage seed weevil were amongst the highest, strongly imply that fodder radish could promote populations of this pest. Indeed, the June suction sample, which most likely contained a proportion of newly emerged parasitoids of cabbage seed weevil, yielded fewer individuals from fodder radish than from the other brassicas, implying that parasitoid production from these plots was low. It is not certain, however, that the sampled parasitoids actually emerged from the plots on which they were found, and the pod dissection data should be treated with caution as the brassicas were sampled on different dates to ensure that growth stages were consistent.

The comparatively high numbers of both adult cabbage stem weevils (*C. pallidactylus*) and parasitoids in the suction samples from fodder radish did not translate into higher numbers of parasitoids emerging from these plots. Most weevil parasitoids emerged from plots of the forage rape cultivars, suggesting that the reproductive success of the parasitoid may be limited on fodder radish. In July, the increase in cabbage stem weevil numbers relative to the two prior sample dates most likely represented emergence of new-generation adults. Interestingly, fodder radish yielded the highest weevil numbers in July, and whilst some individuals may not have emerged from the plots on which they were found, this observation adds weight to the evidence that fodder radish favours production of the pest rather than of the parasitoid.

### 4.3. Banker Plants for Parasitoids of Brassica Pod Midge

Preference of brassica pod midge (*D. brassicae*) for *Brassica* hosts supports the findings of previous studies [56] and their parasitoids behaved similarly, with the highest numbers in forage rape. Fodder radish was little utilised by brassica pod midge or its parasitoids, potentially due to the thickened pod wall or surface waxes [57]. No fodder radish pods were infested by the midge and very few parasitoids of brassica pod midge emerged from former fodder radish plots indicating that this brassica has little banker plant value for natural enemies of this pest.

### 4.4. Banker Plants as Trap Crops for Pests of Oilseed Rape and Implications for Parasitoids

The performance of the turnip rape (*Brassica rapa*) cultivars was consistent with earlier work that has shown turnip rape to be more attractive to pollen beetles at the green bud stage than OSR, and can therefore be useful as a trap crop for this pest in integrated control strategies [29,58]. Both adult beetle and larval numbers were higher early in the season on the turnip rape hybrid ‘Tyfon’ and turnip rape cv ‘Jupiter’ than on the other treatments, including OSR. However, production of emerging parasitoids of pollen beetle from turnip rape-type plots ranked lowest amongst the plants tested and the earlier flowering of these plants led to larger proportions of the larval population escaping parasitisation, a factor which may assist proliferation of this pest. Indeed, modelling of the spatio-temporal dynamics of pollen beetle and its parasitoid *T. heterocerus* in relation to changing landscape and agricultural management practices [59] has suggested that turnip rape trap crop mediated increases in pollen beetle densities are not counterbalanced sufficiently by parasitoid regulation. The April peak in parasitoid numbers on the turnip rape types coincided with their flowering growth stages, and unlike the other brassica types, not the bud stages. As a result many of the larvae present during the bud stages would have been too early to experience peak exposure to the parasitoid *P. interstitialis*, which oviposits into eggs and 1st instar larvae through the bud walls [40], and *T. heterocerus*, which prefers to oviposit into large second instar larvae in open flowers [60] and comprised the majority of the individuals suction-sampled from the *B. rapa* cultivars. However, although relatively high proportions of *T. heterocerus* later emerged from these plots, *P. interstitialis* became the predominant species in the emergence samples, indicating that this species was most important in causing parasitisation despite being in a minority on the plants. *Tersilochus heterocerus* can suffer substantial egg mortality within host larvae [61], and is less competitive than *P. interstitialis* [62], factors which may help explain the lower overall parasitoid return from turnip rape plots, and why *P. interstitialis* became the dominant species emerging in the following season.

The turnip rape-type cultivars, particularly ‘Tyfon’, supported large numbers of cabbage seed weevil adults early in the season, and also hosted more cabbage stem weevil individuals than OSR in March, supporting observations that turnip rape is preferred to OSR by these pests and has potential use as a trap crop [29,63]. However, parasitism rates of cabbage seed weevil larvae were relatively low on these brassicas, as reported in the spring OSR study by Kovács et al. [31] but in contrast to their earlier findings [64]. Pod infestation rates of cabbage seed weevil were low on the turnip rape types compared with fodder radish and forage rape (‘Emerald’ and ‘Hobson’), and this, in combination with the low parasitism rates, suggests that these plants may not promote populations of cabbage seed weevils nor its parasitoids.

The greater abundance of *D. brassicae* adults on the turnip rape hybrid ‘Tyfon’ than OSR indicates its potential use as a trap crop for this pest. However, this preference was not reflected in numbers of the midge’s parasitoids. Proportions of infested ‘Tyfon’ pods were low compared with forage rape cultivars and turnip rape ‘Jupiter’, and they were similar to OSR, but parasitoid return was similar amongst all the plants tested (except fodder radish), implying that ‘Tyfon’ shows relatively low levels of acceptance by the pest for oviposition, and that parasitism rates are relatively high on this plant. The brassica pod midge is multivoltine and can have two or three generations on OSR in the UK [65] and its parasitoids are also probably multivoltine [37]; the two peaks in host and parasitoid numbers on the turnip rape cultivars may reflect earlier initial migration followed by an emerging new generation. It is possible that a second peak also occurred for the other plants tested, but beyond the last suction sample date. A possible downside of the early flowering turnip rape cultivars is thus that they may promote the earlier emergence of new-generation pod midge adults, a lower proportion of which is likely to go into diapause [66,67], meaning that larger populations may be available to infest crops later in the season.

### 4.5. Selection of Banker Plants for Pest Management in Oilseed Rape

Despite the absence of useful trap cropping characteristics, the forage rape cultivars, particularly ‘Hobson’, showed good potential as a banker plant for pollen beetle parasitoids. Parasitoid production from former forage rape ‘Hobson’ plots was not significantly lower than from fodder radish (the best for this pest), and parasitism rates of cabbage seed weevil larvae and production of cabbage stem weevil parasitoids were also high on the forage rapes tested. The fact that these brassicas, unlike fodder radish or turnip rape, do not promote populations of any of the OSR pests sampled suggests that forage rape might be the best choice as a banker plant of the six species tested. Although we ran preliminary field trials over two previous years, the data from this study derive from only one field season; further studies would help to support these findings—although as work by Kovács et al. [31] shows, it is probably more important to test such systems in different regions, as the parasitoid composition as well as relative phenology of plants and parasitoids is likely to differ with region/country.

A range of brassicas (often fodder radish and/or forage rape) are sometimes included in the bird seed field margin mixtures currently prescribed under European agri-environment schemes [68]. Our results suggest that more careful consideration of the brassica component of these mixtures could extend margin functionality towards enhanced biocontrol of specialist pests and help avoid their exacerbation. If fodder radish or the turnip rape types are to be considered, a careful assessment will need to be made of the relative importance of the pests in the local area.

This work also highlights the importance of investigating potential effects of the plants considered for field margin mixtures on the entire suite of pests associated with a particular crop rotation. As the example of fodder radish illustrates, focusing on the positive aspects for one major pest alone risks elevating the status of others. Indeed, similar phenomena could apply to other systems. For example, OSR is often grown in rotation with field beans (*Vicia faba* L.) and the leguminous plants included in current pollen and nectar field margin mixtures [69] could differentially influence populations of the specialist weevil (*Sitona lineatus* L.) and bruchid pests (*Bruchus rufimanus* Boh.) of the crop, and those of their respective parasitoid complexes.

## 5. Conclusions

Wildflower margins sown as strips within the crop or at the field margin are becoming commonplace in European agriculture. Careful design of the composition of flower mixtures could help improve the multifunctionality of these agri-environmental interventions, especially in relation to pest control of specialist pests with specialist natural enemies. Pests and their parasitoids responded differently to the six potential banker plants tested in this study. Fodder radish (*Raphanus sativus*) was the best species for control of pollen beetle (the main pest of OSR at the inflorescence stage) as it had potential as a trap crop and best supported pollen beetle parasitoids, but this species did not support well the parasitoids of weevil pests (*Ceutorhynchus obstrictus* and *C. pallidactylus*) and may therefore proliferate these pests. This highlights the dangers of a focussed study on a single pest and the whole pest–natural enemy complex should therefore be considered wherever possible. Forage rape (*Brassica napus* L. subsp. Biennis), especially cv ‘Hobson’, proved to be the best all-rounder, supporting parasitoids of all pests studied. Further work needs to be performed testing these plants in multiple years in several locations where the pest–parasitoid complex may be different. The principles of this study could be applied to other specialist crop plant–pest–parasitoid systems.

## Figures and Tables

**Table 2:** Whole-season means (log scale), and underneath in brackets back transformed means, of oilseed rape pests (white rows) and their parasitoids (shaded rows) collected by suction sampling from plots (*n* = 4) of different Brassicaceae: Fodder radish (*Raphanus sativus*) cv Apoll; Oilseed rape (OSR; *Brassica napus*) cv Castille; Forage rape (*Brassica napus*) cv Emerald and cv Hobson; Turnip rape (*Brassica rapa*) cv Jupiter and Tyfon (a hybrid of *B. rapa* Rapifer group × *B. rapa* Pekinensis group). Asterisks (*) indicate use of an offset. Smallest population underscored, largest population bold.

	Fodder Radish	OSR	Forage Rape ‘Emerald’	Forage Rape ‘Hobson’	Turnip Rape ‘Jupiter’	Tyfon	F_5,15_	*P*	LSD
Pollen beetle (*Brassicogethes aeneus*) (adults)	**2.790** **(616.6)**	2.458 (287.1)	2.622 (418.8)	2.635 (431.5)	2.628 (424.6)	2.725 (530.9)	1.74	0.186	0.257
Pollen beetle (larvae)	1.865 (73.3)	1.578 (37.8)	1.655 (45.2)	1.890 (77.6)	1.753 (56.6)	**2.216** **(164.4)**	9.96	<0.001	0.215
Parasitoids of pollen beetle	**2.388** **(244.3)**	1.716 (52.0)	1.924 (83.9)	1.909 (81.1)	1.686 (48.5)	1.647 (44.4)	6.38	0.002	0.329
Cabbage seed weevil (*Ceutorhynchus obstrictus*) adults	0.527 (3.4)	0.931 (8.5)	0.389 (2.4)	0.615 (4.1)	1.033 (10.8)	**1.330** **(21.4)**	14.04	<0.001	0.286
Parasitoids of cabbage seed weevil	0.940 (8.7)	**1.353** **(22.5)**	1.216 (16.4)	1.299 (19.9)	1.065 (11.6)	1.315 (20.7)	5.13	0.006	0.217
Brassica pod midge (*Dasineura brassicae*) adults *	0.815 (5.5)	1.657 (44.4)	1.906 (79.5)	1.691 (48.1)	1.418 (25.2)	**2.157** **(142.5)**	11.08	0.006	0.418
Parasitoids of brassica pod midge	0.913 (8.2)	1.500 (31.6)	**1.827** **(67.1)**	1.647 (44.4)	0.993 (9.8)	1.457 (28.6)	14.85	<0.001	0.284
Cabbage stem weevil (*Ceutorhynchus pallidactylus*) adults	**1.692** **(49.2)**	0.714 (5.2)	0.935 (8.6)	1.089 (12.3)	1.330 (21.4)	1.221 (16.6)	19.64	<0.001	0.230
Parasitoids of cabbage stem weevil *	**1.174** **(13.9)**	0.556 (2.6)	0.336 (1.2)	0.345 (1.2)	0.389 (1.4)	0.301 (1.0)	7.65	<0.001	0.364

## Data Availability

Data are freely available upon reasonable request.

## Supplementary Materials

The following supporting information can be downloaded at: https://www.mdpi.com/article/10.3390/insects14040349/s1, Table S1. Brassica type x time interaction results from repeated measures ANOVA of monthly suction sample totals for insect species collected using a vortis suction sampler from replicated plots (*n* = 4) of six different Brassicaceae: Fodder radish (*Raphanus sativus*) cv Apoll, Oilseed rape (OSR; *Brassica napus*) cv Castille, Forage rape (*Brassica napus*) cv Emerald and Hobson, Turnip rape (*Brassica rapa*) cv Jupiter, and Tyfon (a hybrid of *B. rapa* Rapifer group × *B. rapa* Pekinensis group); Table S2. Summary of the main responses of pests of oilseed rape and their parasitoids to tested plants of the Brassicaceae: Fodder radish (*Raphanus sativus*) cv Apoll, Oilseed rape (OSR; *Brassica napus*) cv Castille, Forage rape (*Brassica napus*) cv Emerald and Hobson, Turnip rape (*Brassica rapa*) cv Jupiter, and Tyfon (a hybrid of *B. rapa* Rapifer group × *B. rapa* Pekinensis group); Figure S1. Species composition of the parasitoids of (a) Pollen beetle (*Brassicogethes aeneus*), (b) Cabbage seed weevil (*Ceutorhynchus obstrictus*), and (c) Brassica pod midge (*Dasineura brassicae*) collected in suction samples and totalled from plots (*n* = 4) of different Brassicaceae: Fodder radish (*Raphanus sativus*) cv Apoll, Oilseed rape (OSR; *Brassica napus*) cv Castille, Forage rape (*Brassica napus*) cv Emerald and Hobson, Turnip rape (*Brassica rapa*) cv Jupiter, and Tyfon (a hybrid of *B. rapa* Rapifer group × *B. rapa* Pekinensis group). Parasitoids: [*Tersilochus heterocerus* Thoms., *Phradis interstitialis* Thoms. *Phradis morionellus* Holm; *Diospilus capito* (Nees), *Blacus nigricornis* (Haeselbarth); *Stenomalina gracilis* (Walker), *Mesopolobus morys* (Walker), *Trichomalus perfectus* (Walker); *Platygaster subuliformis* (Kieffer); *Omphale clypealis* (Thompson)]; Figure S2. Species composition of the hymenopteran parasitoids of (a) Pollen beetle (*Brassicogethes aeneus*), (b) Brassica pod midge (*Dasineura brassicae*) collected in total in emergence trap samples from the fallow in the positions of former brassica plots (i.e., those insects emerging from the soil the following year after dropping from their host plant to pupate). Host brassicas tested in plots (*n* = 4) comprising: Fodder radish (*Raphanus sativus*) cv Apoll, Oilseed rape (OSR; *Brassica napus*) cv Castille, Forage rape (*Brassica napus*) cv Emerald and Hobson, Turnip rape (*Brassica rapa*) cv Jupiter and Tyfon (a hybrid of *B. rapa* Rapifer group × *B. rapa* Pekinensis group). Parasitoids: [*Tersilochus heterocerus* Thoms., *Phradis interstitialis* Thoms. *Phradis morionellus* Holm; *Diospilus capito* (Nees), *Blacus nigricornis* (Haeselbarth); *Platygaster subuliformis* (Kieffer); *Omphale clypealis* (Thompson)].
