# Supplementary material for: Banker Plant Bonuses? The Benefits and Risks of Including Brassicas in Field Margins to Promote Conservation Biocontrol of Specialist Pests in Oilseed Rape
Source: Insects. 2023 Mar 31;14(4):349. doi: 10.3390/insects14040349 (PMC10145157; doi:10.3390/insects14040349)
Supplement: Supplementary file 1 [file insects-14-00349-s001.zip › insects-2242559-supplementary.pdf]

Banker plant bonuses? The benefits and risks of including plants in field margins to promote conservation biocontrol of specialist pests

Matthew. P. Skellern, Suzanne J. Clark, Andrew W. Ferguson, Nigel P. Watts & Samantha M. Cook*

* Corresponding author: Samantha M. Cook, Rothamsted Research, Harpenden, Hertfordshire, AL5 2JQ, UK

Email: sam.cook@rothamsted.ac.uk

Supplementary Materials

Table S1. Brassica type x time interaction results from repeated measures ANOVA of monthly suction sample totals for insect species collected using a vortis suction sampler from replicated plots (*n*=4) of six different Brassicaceae: Fodder radish (*Raphanus sativus*) cv Apoll, Oilseed rape (*Brassica napus*) cv Castille, Forage rape (*Brassica napus*) cv Emerald and Hobson, Turnip rape (*Brassica rapa*) cv Jupiter, and Tyfon (a hybrid of B. *rapa* Rapifer group x *B. rapa* Pekinensis group).

Captured insects	Degrees of freedom	F	*P*-value
Pollen beetle (*Brassicogethes aeneus*) adults	16.7, 60.0	5.13	<0.001
Pollen beetle larvae	10.5, 37.7	13.71	<0.001
Pollen beetle parasitoids	10.0, 36.1	11.95	<0.001
Cabbage seed weevil (*Ceutorhynchus obstrictus*) adults	9.2, 33.2	3.97	0.002
Cabbage seed weevil parasitoids	16.7, 60.0	5.77	<0.001
Brassica pod midge (*Dasineura brassicae*) adults	13.4, 48.4	9.37	<0.001
Brassica pod midge parasitoids	13.9, 50.2	5.75	<0.001
Cabbage stem weevil (*Ceutorhynchus pallidactylus*) adults	12.5, 45.1	8.68	<0.001
Cabbage stem weevil parasitoids	8.2, 29.3	5.34	<0.001

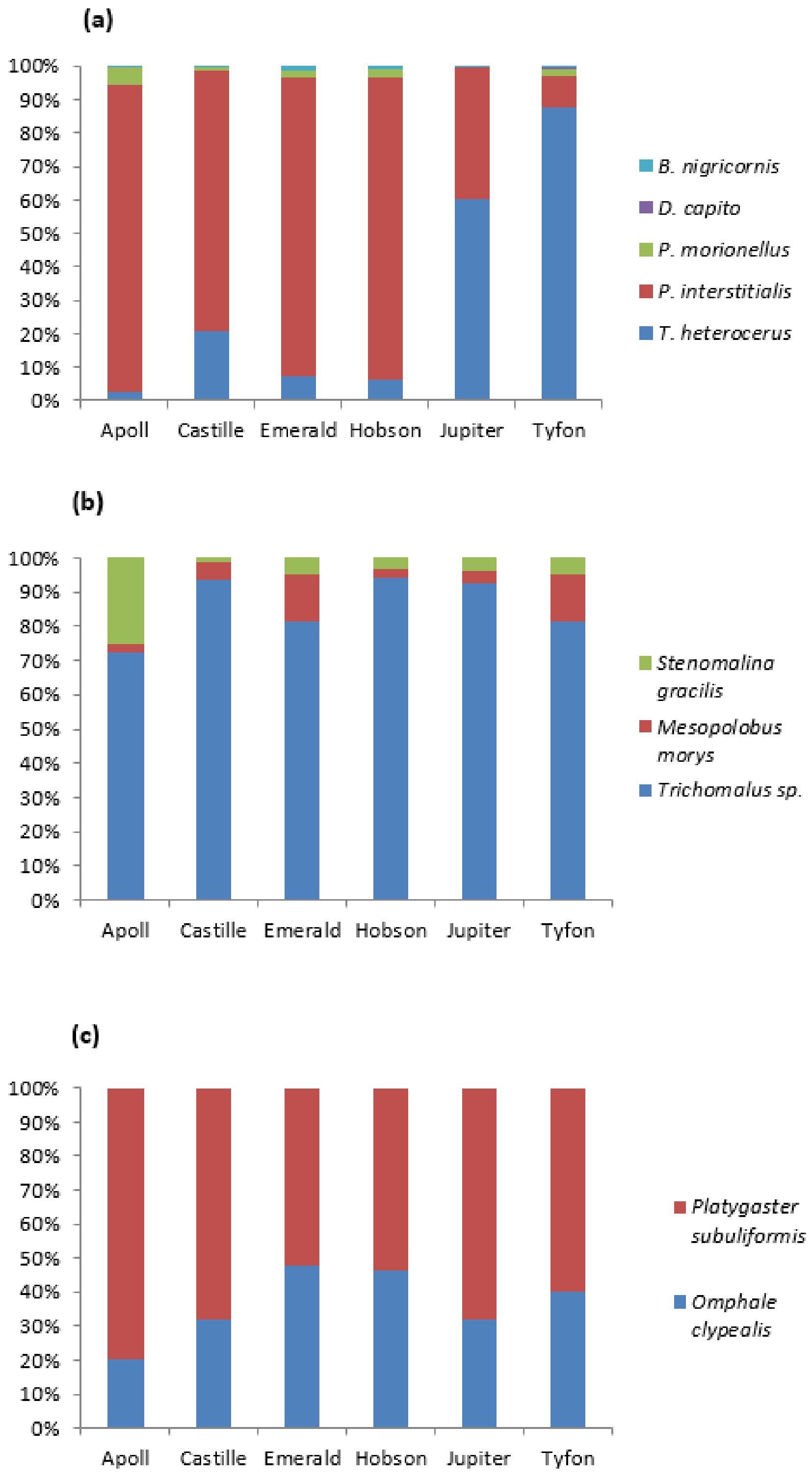

Figure S1. Species composition of the parasitoids of (a) Pollen beetle (*Brassicogethes aeneus*), (b) Cabbage seed weevil (*Ceutorynchus obstrictus*), and (c) Brassica pod midge (*Dassineura brassicae*) collected in suction samples and totalled from plots (*n*=4) of different Brassicaceae: Fodder radish (*Raphanus sativus*) cv Apoll, Oilseed rape (*Brassica napus*) cv Castille, Forage rape (*Brassica napus*) cv Emerald and Hobson, Turnip rape (*Brassica rapa*) cv Jupiter, and Tyfon (a hybrid of *B. rapa* Rapifer group x *B. rapa* Pekinensis group). Parasitoids: [*Tersilochus heterocerus* Thoms., *Phradis interstitialis* Thoms. *Phradis morionellus* Holm; *Diospilus capito* (Nees), *Blacus nigricornis* (Haeselbarth); Stenomalina gracilis (Walker), *Mesopolobus morys* (Walker), *Trichomalus perfectus* (Walker); Platygaster subuliformis (Kieffer); Omphale clypealis (Thompson)].

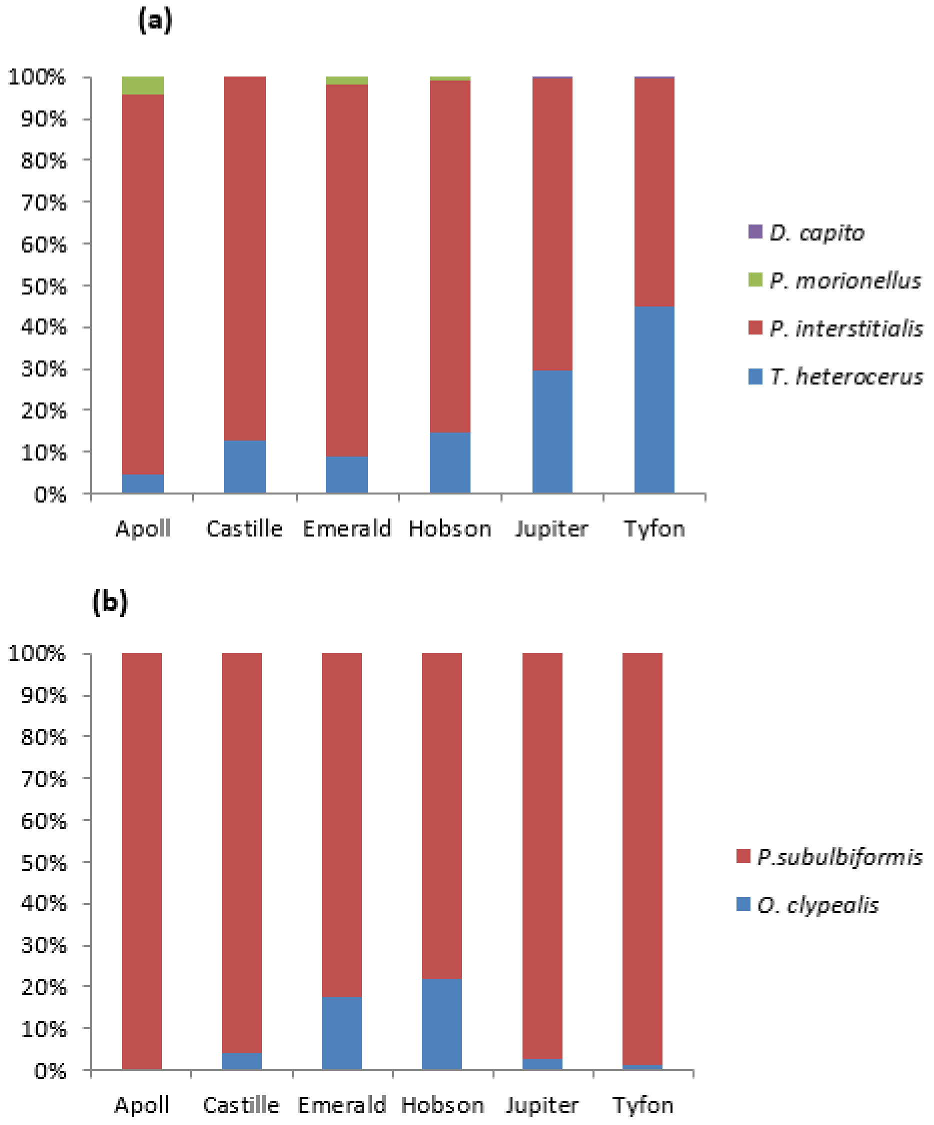

Figure S2. Species composition of the hymenopteran parasitoids of (a) Pollen beetle (*Brassicogethes aeneus*), (b) Brassica pod midge (*Dasineura brassicae*) collected in total in emergence trap samples from the fallow in the positions of former brassica plots (i.e. those insects emerging from the soil the following year after dropping from their host plant to pupate). Host brassicas tested in plots (*n*=4) comprising: Fodder radish (*Raphanus sativus*) cv Apoll, Oilseed rape (*Brassica napus*) cv Castille, Forage rape (*Brassica napus*) cv Emerald and Hobson, Turnip rape (*Brassica rapa*) cv Jupiter and Tyfon (a hybrid of *B. rapa* Rapifer group x *B. rapa* Pekinensis group). Parasitoids: [*Tersilochus heterocerus* Thoms., *Phradis interstitialis* Thoms. *Phradis morionellus* Holm; *Diospilus capito* (Nees), *Blacus nigricornis* (Haeselbarth); *Platygaster subuliformis* (Kieffer); *Omphale clypealis* (Thompson)].

Table S2. Summary of the main responses of pests of oilseed rape and their parasitoids sampled to tested plants of the Brassicaceae: Fodder radish (*Raphanus sativus*) cv Apoll, Oilseed rape (OSR; *Brassica napus*) cv Castille, Forage rape (*Brassica napus*) cv Emerald and Hobson, Turnip rape (*Brassica rapa*) cv Jupiter, and Tyfon (a *B. rapa* hybrid).

Pest / Parasitoid	Highest abundance*	Lowest abundance*
Pollen beetle (*Brassicogethes aeneus*) adults (suction sample from plots)	Fodder radish	OSR
Pollen beetle larvae (suction sample from plots)	Tyfon	OSR
Pollen beetle parasitoids (suction sample from plots)	Fodder radish	Tyfon
Pollen beetle parasitoids (emerged from treatment plots)	Fodder radish	Tyfon
Cabbage seed weevil (*Ceutorhynchus obstrictus*) adults (suction sample from plots)	Tyfon	Forage rape
Cabbage seed weevil parasitoids (suction sample from plots)	OSR	Fodder radish
Cabbage seed weevil larvae (pod samples)	Forage rape	Tyfon
Parasitized cabbage seed weevil larvae (pod samples)	OSR = Forage rape	Fodder radish (0)
Cabbage seed weevil parasitoids (emerged from treatment plots)	N/A (parasitoids emerge directly from pods and do not drop to the ground to pupate in the soil)	
Cabbage stem weevil (*Ceutorhynchus pallidactylus*) adults (suction sample from plots)	Fodder radish	OSR
Cabbage stem weevil parasitoids (suction sample from plots)	Fodder radish	Tyfon
Cabbage stem weevil (parasitoids (emerged from treatment plots)	Forage rape	Tyfon
Brassica pod midge (*Dasineura brassicae*) adults	Tyfon	Fodder radish
Brassica pod midge parasitoids	Forage rape	Fodder radish
Cabbage stem weevil (*Ceutorhynchus pallidactylus*) adults	Forage rape	Fodder radish (0)
Cabbage stem weevil parasitoids	Forage rape	Fodder radish

* irrespective of statistical significance
